# Shade signals alter the expression of circadian clock genes in newly‐formed bioenergy sorghum internodes

**DOI:** 10.1002/pld3.235

**Published:** 2020-06-25

**Authors:** Tesfamichael H. Kebrom, Brian A. McKinley, John E. Mullet

**Affiliations:** ^1^ Cooperative Agricultural Research Center College of Agriculture and Human Sciences Prairie View A&M University Prairie View TX USA; ^2^ Center for Computational Systems Biology College of Engineering Prairie View A&M University Prairie View TX USA; ^3^ Department of Biochemistry and Biophysics Texas A&M University College Station TX USA

**Keywords:** bioenergy, circadian clock, internode, shade, sorghum, stem, transcriptome

## Abstract

Stem internodes of bioenergy sorghum inbred R.07020 are longer at high plant density (shade) than at low plant density (control). Initially, the youngest newly‐formed subapical stem internodes of shade‐treated and control plants are comparable in length. However, full‐length internodes of shade‐treated plants are three times longer than the internodes of the control plants. To identify the early molecular events associated with internode elongation in response to shade, we analyzed the transcriptome of the newly‐formed internodes of shade‐treated and control plants sampled between 4 and 6 hr after the start of the light period (14 hr light/10 hr dark). Sorghum genes homologous to the Arabidopsis shade marker genes *ATHB2* and *PIL1* were not differentially expressed. The results indicate that shade signals promote internode elongation indirectly because sorghum internodes are not illuminated and grow while enclosed with leaf sheaths. Sorghum genes homologous to the Arabidopsis morning‐phased circadian clock genes *LHY*, *RVE*, and *LNK* were downregulated and evening‐phased genes such as *TOC1*, *PRR5,* and *GI* were upregulated in young internodes in response to shade. We hypothesize that a change in the function or patterns of expression of the circadian clock genes is the earliest molecular event associated with internode elongation in response to shade in bioenergy sorghum. Increased expression of *CycD1*, which promotes cell division, and decreased expression of cell wall‐loosening and *MBF1*‐like genes, which promote cell expansion, suggest that shade signals promote internode elongation in bioenergy sorghum in part through increasing cell number by delaying transition from cell division to cell expansion.

## 1 INTRODUCTION

Bioenergy sorghum accumulates ~80% of its biomass in its stem (McKinley, Olson, et al., 2018). One of the primary biological functions of the stem is the storage of carbohydrates such as sucrose and starch (McKinley, Casto, Rooney, & Mullet, 2018). The capacity of sorghum stems for biomass and sugar accumulation is correlated with its length and girth (Murray, Rooney, et al., 2008; Murray, Sharma, et al., 2008; Ritter et al., 2008). Because of these desirable attributes, the stem is a target for improving bioenergy crops such as sorghum, maize, switchgrass, and sugarcane that are grown for biomass for forage, biofuel feedstock, or sugars for human consumption (Slewinski, 2012). The large biomass and sugar yield of these species is in part due to their utilization of C4 photosynthesis (Mullet, 2017). Because of its genetic diversity, adaptation to diverse climates, and small sequenced genome, sorghum is a model plant for studying stem growth, biomass accumulation, and sugar production in C4 crops (Mullet et al., 2014; Slewinski, 2012).

The stems of grasses are formed as a series of internodes generated by the activities of the lower section of the shoot apical meristem known as the rib zone (McKim, 2019; Serrano‐Mislata & Sablowski, 2018). Internodes in bioenergy sorghum develop during the vegetative stage and further elongate when grown at high plant density where plants shade each other (Kebrom, McKinley, & Mullet, 2017). Shade signals also inhibit shoot branching and promote early flowering (Smith & Whitelam, 1997). These responses are collectively known as the shade avoidance syndrome (SAS) (Casal, 2013; Smith & Whitelam, 1997). The elongation of the stem of a plant in response to shade by its neighbors elevates the leaves out of shade to get sunlight for photosynthesis. Understanding how shade signals modulate stem elongation in bioenergy sorghum will be useful to increase the biomass and sugar yield of crops through agronomy, breeding, and genetic manipulation of the size of internodes.

Plants use photoreceptors to monitor their light environment and proximity to potential competitors for light. Plant photoreceptors that detect neighbor proximity and shade signals include the red (R) and far‐red (FR) light absorbing phytochromes, blue light absorbing cryptochromes, and UV‐B absorbing UVR8 (Ballare & Pierik, 2017). Research on shade signaling has focused on phytochromes because of the ability of this small family of photoreceptors, encoded by up to five genes (PhyA to PhyE) in Arabidopsis and three genes in the grasses (PhyA, PhyB, and PhyC), to detect neighbor proximity and shade signals before a plant is completely shaded by its neighbors (Ballare & Pierik, 2017; Ballare, Scopel, & Sanchez, 1990; Mathews & Sharrock, 1996). Besides their role in shade signaling, phytochromes regulate many other aspects of plant growth and development, including seed germination and flowering time (Franklin et al., 2003; Franklin & Whitelam, 2004). Each phytochrome may play a major role at specific stages during plant growth and development. The shoot elongation response to shade signals is mediated mainly through the activities of phytochrome B (PhyB) (Franklin & Quail, 2010; Martinez‐Garcia et al., 2010), which is localized in the cytoplasm in a R light absorbing inactive Pr form. Once it is activated by R light, PhyB is converted into a FR absorbing Pfr form, moves into the nucleus, and regulates the expression and activities of genes to modulate plant growth and development.

Plants absorb R light for photosynthesis and transmit or reflect FR light. At high plant density, the absorption of R and reflection of FR reduces the R‐to‐FR ratio. At low R:FR, PhyB is in the inactive R light absorbing Pr form, which signals plants to anticipate shading by their neighbors and thus initiate the shade avoidance developmental program including shoot elongation. When leaves are exposed to full sunlight, PhyB is in its active FR light absorbing Pfr form and shoot elongation is suppressed. Therefore, the proportion of Pr and Pfr form of PhyB in plants is proportional to the level of R and FR illumination in their microenvironment, and the extent of shoot elongation (Casal, 2013). In PhyB‐deficient mutant plants, the shoot elongation developmental program is activated in all environments. The molecular mechanisms promoting shoot elongation in response to low R:FR lights have been investigated in more detail in Arabidopsis seedlings (reviewed in Ballare & Pierik, 2017; Fiorucci & Fankhauser, 2017; Wang & Wang, 2015).

At high R:FR, active PhyB inhibits hypocotyl elongation in Arabidopsis seedlings by inactivating members of the bHLH family of transcription factors known as Phytochrome Interacting Factors (PIFs) (Leivar & Quail, 2011). PIFs promote hypocotyl elongation under low R:FR lights in part by inducing the expression of auxin biosynthesis genes (Hornitschek et al., 2012; Li et al., 2012; Lorrain, Allen, Duek, Whitelam, & Fankhauser, 2008; Muller‐Moule et al., 2016). Auxin enhances the expression of cell wall loosening genes such as *xyloglucan endotransglucosylase hydrolases* (*XTHs*) (Sasidharan et al., 2010). Cell wall loosening genes facilitate cell expansion and, thus, shoot elongation. Several other bHLH transcription factors such as *HFR1*, *PAR1,* and *PAR2* are upregulated by shade signals, and prevent PIFs from promoting excessive shoot elongation in response to shade (Galstyan, Cifuentes‐Esquivel, Bou‐Torrent, & Martinez‐Garcia, 2011; Roig‐Villanova et al., 2007; Sessa et al., 2005). Genes encoding HD‐ZIP transcription factors that are upregulated in response to shade include *ATHB2*, *ATHB4*, and *HAT2* (Roig‐Villanova, Bou, Sorin, Devlin, & Martinez‐Garcia, 2006). The expression of the Arabidopsis *HFR1*, *PAR,* and *ATHB2* genes is rapidly induced by shade, and these genes serve as markers for the perception of shade signals (Martinez‐Garcia et al., 2010; Procko, Crenshaw, Ljung, Noel, & Chory, 2014).

Besides phytochromes, blue light (B) photoreceptors cryptochromes (CRY1 and CRY2) and UV‐B photoreceptor UVR8 also mediate response to neighbor proximity and shade signals (Fiorucci & Fankhauser, 2017). B light is low under plant canopies and induces shade avoidance responses (Keuskamp et al., 2011). CRY2 interacts with PIF4 and PIF5 to promote hypocotyl elongation in response to low blue light (Pedmale et al., 2016). All the three types of plant photoreceptors also regulate hypocotyl growth by controlling the CONSTITUTIVELY PHOTOMORPHOGENIC 1 (COP1)/SUPPRESSOR of PhyA‐105 (SPA) complex. The COP1/SPA ubiquitin E3 ligase complex represses photomorphogenesis and promotes hypocotyl elongation in the dark by inactivating the bZIP transcription factor ELONGATED HYPOCOTYL 5 (HY5) (Pacin, Legris, & Casal, 2014). Light‐activated phytochromes and cryptochromes interact with SPA, and UV‐B‐activated UVR8 interact with COP1 to prevent the COP1/SPA complex from inactivating HY5 (Huang et al., 2013; Lian et al., 2011; Liu, Zuo, Liu, Liu, & Lin, 2011; Lu et al., 2015; Sheerin et al., 2015). HY5 regulates the expression of other genes to inhibit hypocotyl elongation and promote photomorphogenesis (Abbas, Maurya, Senapati, Gangappa, & Chattopadhyay, 2014; Zhao et al., 2019). HY5 is also involved in shade signaling possibly as a repressor of shoot elongation (Ciolfi et al., 2013; Nozue et al., 2015; Sellaro, Yanovsky, & Casal, 2011). Furthermore, the expression of *HY5* in the root increases in response to low R:FR perception by the shoot, and HY5 reduces lateral root growth (van Gelderen et al., 2018).

Plant hormones, such as auxin, gibberellic acid (GA), ethylene, and brassinosteroids (BRs), promote elongation of hypocotyls and seedlings of Arabidopsis and other plant species (Kozuka et al., 2010; Nozue & Maloof, 2006; Stamm & Kumar, 2010). Plant photoreceptors directly or indirectly control the level and/or activities of these plant hormones to promote or inhibit shoot elongation (Ballare & Pierik, 2017; Fiorucci & Fankhauser, 2017). Plant photoreceptors also play a role in the circadian clock–regulated plant growth and developmental processes (Devlin & Kay, 2001). The plant circadian clock has three components: inputs that reset the clock, core clock oscillator, and outputs such as physiological, metabolic, and developmental processes (Greenham & McClung, 2015; Hsu & Harmer, 2014; Sanchez & Kay, 2016). Light perceived by phytochromes, cryptochromes and UVR8 resets the circadian clock, by regulating the expression or activities of core clock genes such as *CIRCADIAN CLOCK ASSOCIATED 1* (*CCA1*) and *LATE ELONGATED HYPOCOTYL* (*LHY*), to synchronize plant physiological processes with the day/night cycles (Oakenfull & Davis, 2017; Somers, Devlin, & Kay, 1998; Wenden et al., 2011). Attenuation of the quality or intensity of light might change the timing of expression or activities of the core clock genes and promote or inhibit hypocotyl or shoot elongation (Wenden et al., 2011).

Although research on shoot elongation in response to shade has focused mainly on Arabidopsis seedlings, shade signals in crops growing at high plant density in the field arise in adult plants as neighbor proximity increases with higher planting density and enlargement of canopy size during development. Therefore, to identify the earliest molecular mechanisms regulating the response to shade signals in adult plants, we investigated the shoot elongation response of bioenergy sorghum inbred line R.07020 to high plant density treatments beginning at 60 days after planting. Our study identified differential regulation of core clock and clock‐associated genes in newly‐formed subapical internodes indicating that the circadian clock plays a role in shade‐induced internode elongation in adult plants. The elongating stem tissues in sorghum are enclosed by several layers of leaves and sheaths and are not exposed to direct light. Therefore, we hypothesize that shade signals indirectly regulate the expression of clock genes and promote internode elongation in sorghum. The results highlight the need for more research on the role of the circadian clock in shade signaling, stem growth, and biomass accumulation in adult plants.

## 2 RESULTS

### 2.1 Response of bioenergy sorghum inbred R.07020 plants to high plant density (shade)

**FIGURE 1:**
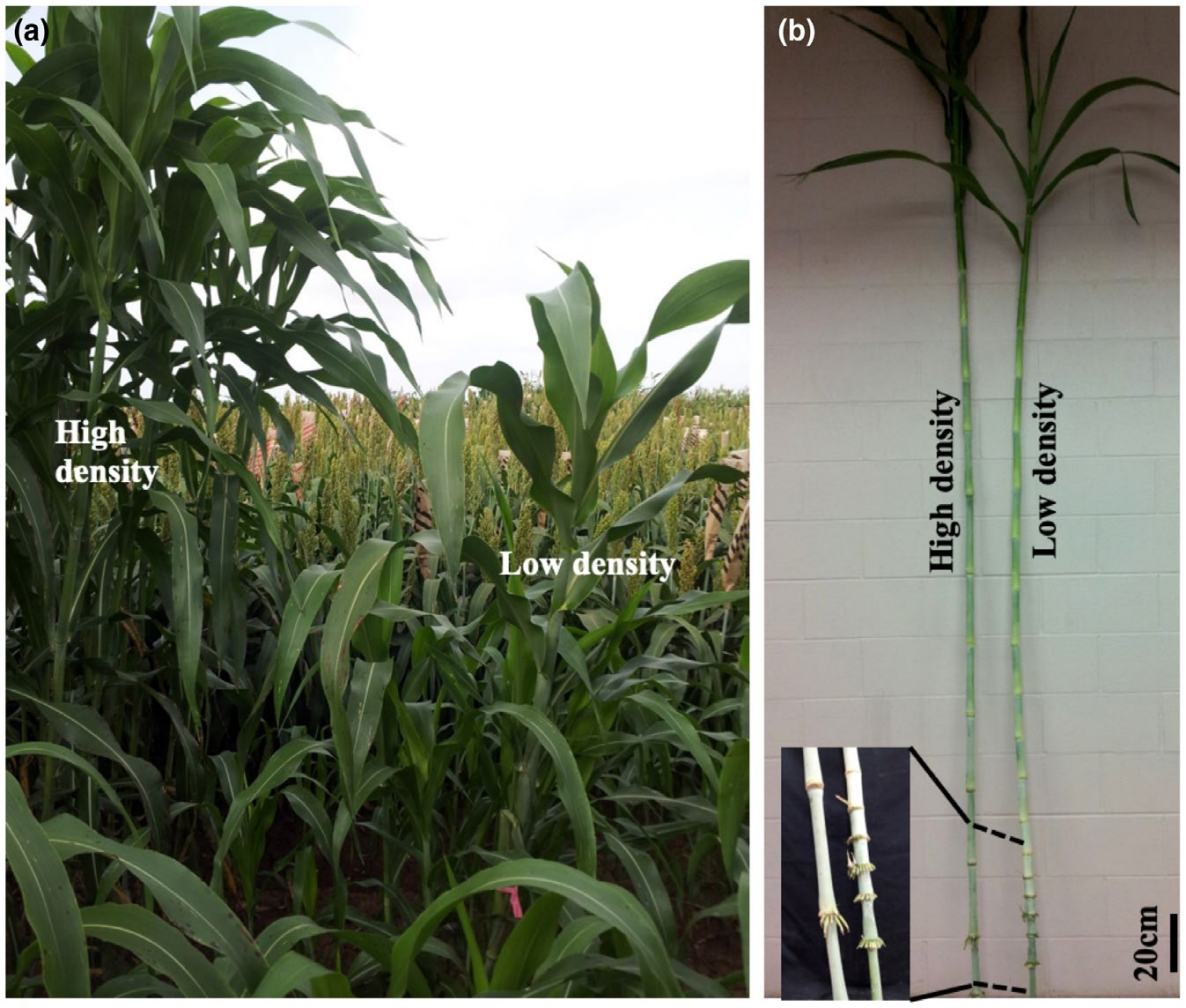
Internode elongation in response to high plant density (shade) in bioenergy sorghum genotype R.07020. (a) R.07020 plants were grown in the field at high density. During early vegetative stage, the surrounding shoots of some of the plants were trimmed to ground level to reduce plant density (low density). Subsequently the height of plants at low density was reduced due to inhibition of internode elongation. (b) The plants at low density eventually elongated as the plant density increased due to regrowth of shoots from the stubble and their plant height was similar to the plants at high density. A series of short internodes between elongated internodes (inset, b) in the stem of plants from low density indicates inhibition of internode growth in R.07020 at low density

Stem internodes of the bioenergy sorghum inbred line R.07020 are formed during the vegetative phase and elongate in response to high plant density or shade signals (Kebrom et al., 2017). As shown in Figure 1, internodes elongated when the plants were grown at high density in the field. When plant density was reduced by trimming some of the shoots to ground level leaving a solitary shoot, internode elongation was suppressed. Regrowth of new shoots from the stubble increased plant density and promoted elongation of newly‐formed internodes in the solitary shoot. A series of short internodes between elongated internodes of the stem of the solitary shoot indicates that the final length of each internode is determined by prevailing growing conditions during its developmental window.

**FIGURE 2:**
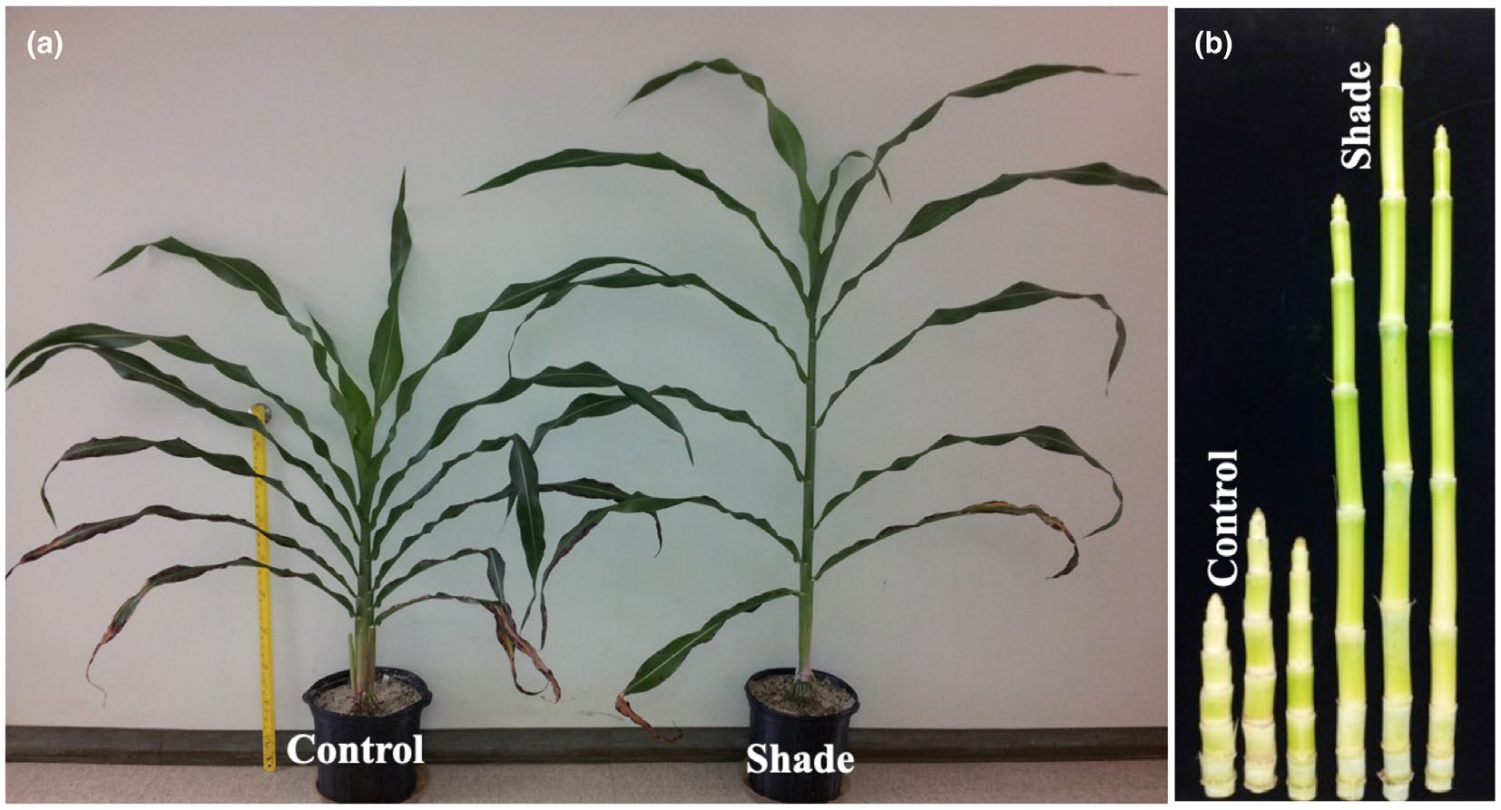
Shoot and stem growth of of bioenergy sorghum genotype R.07020 grown at low plant density (control) and high plant density (shade). Shade treatment (crowding potted plants) was started at 60 days after planting and the plants were photographed after two weeks. Dry lower leaves were removed from the plants before photographing

**FIGURE 3:**
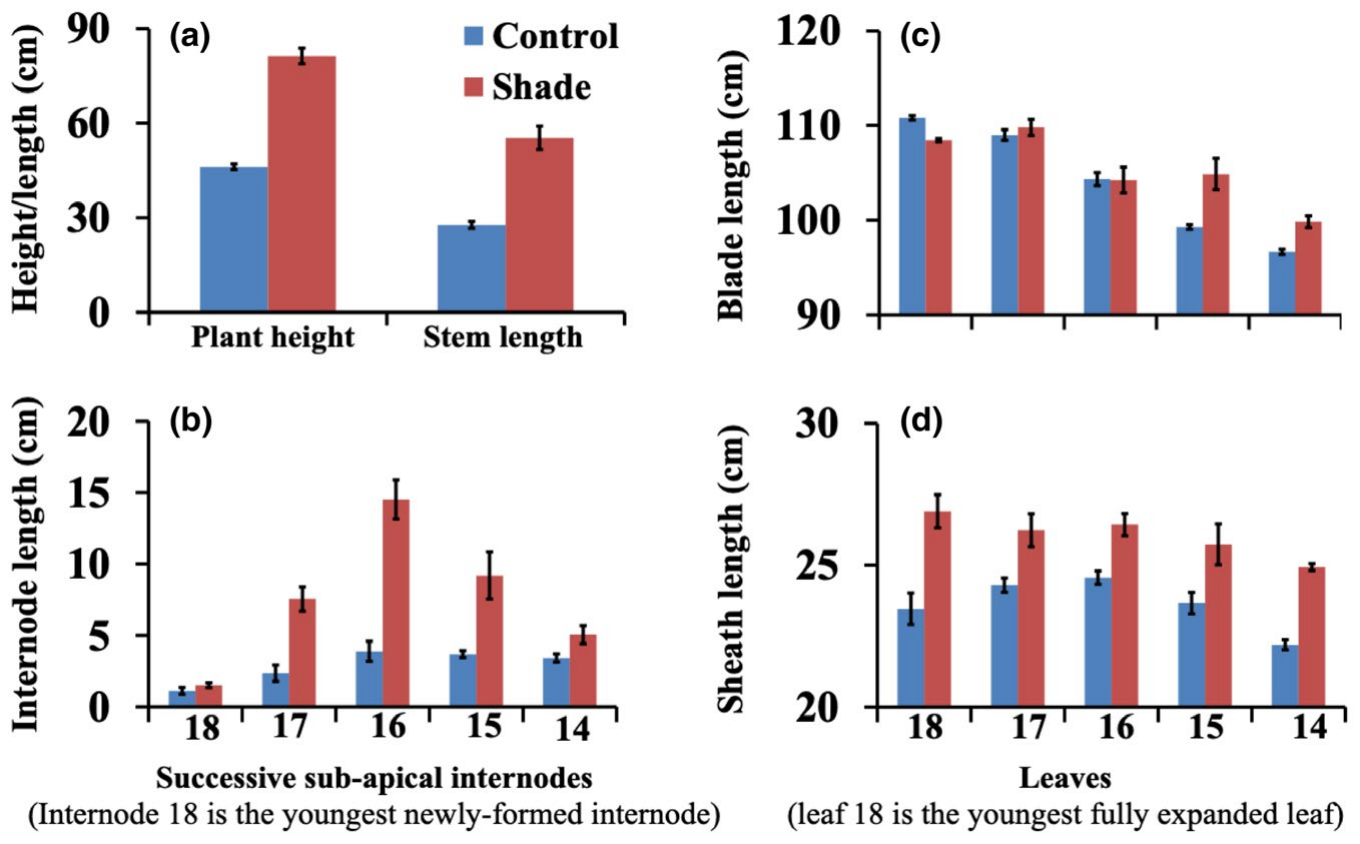
Response of bioenergy sorghum genotype R.07020 to shade. R.07020 plants were grown in growth chambers in pots at low density until 60 days after planting (60 DAP). A set of plants were crowded for the next ten days by bringing the pots closer to simulate a high plant density growing conditions (shade). At sampling (70 DAP), the control and shade treated plants developed 18 fully expanded leaves from 14 fully expanded leaves at 60 DAP. (a) Plant height (from the base of the plant to the top most ligule) and stem length. (b) Length of the five successive sub‐apical internodes, internode 18 is the youngest newly‐formed sub‐apical internode corresponding to the youngest fully expanded leaf (leaf 18) at the time of sampling. (c) Leaf blade, and (d) leaf sheath lengths of the youngest five fully expanded leaves. Data are mean ± SE; *N* = 5

The response of R.07020 plants to high plant density (shade) was investigated in detail by growing plants in pots in growth chambers in a 3.5 m^2^ growth area. The plants were grown in two growth chambers at low density with ample space between plants to avoid mutual shading. Tillers were also removed as they appeared in order to reduce shading. At 60 days after planting (DAP) the plants developed 14 fully expanded leaves. A set of plants in the first growth chamber were maintained at low plant density (control) by reducing the number of plants from 12 to 6, with no physical contact between the plants. The six potted plants from the first growth chamber were transferred to the second growth chamber and thus the number of plants in the second growth chamber was increased from 12 to 18 (shade). In addition, the potted plants in the second growth chamber were crowded by bringing the pots closer to enhance mutual shading. As shown in Figure 2, at 14 days after the onset of the shade treatment (74 DAP) the plants in the shade treatment were taller than those in the control that continued to grow at low plant density. At 10 days after the onset of shade treatment (70 DAP), both control and shade‐treated plants developed 18 fully expanded leaves. The average plant height of the shade‐treated and control plants, from the soil surface to the collar of the youngest fully expanded leaf, was 81.3 cm and 46.2 cm, respectively (Figure 3a). The average stem length was 55.3 cm in the shade‐treated plants and 27.7 cm in the control (Figure 3a). The average length of the youngest newly‐formed internode below the shoot apex in shade‐treated plants was not different from the corresponding internode in the control (Figure 3b). The three internodes below the first subapical internodes in shade‐treated plants were significantly longer than the corresponding internodes in the control. The length of the fifth internode below the shoot apex in shade‐treated plants was slightly longer but not significantly different from the corresponding internode in the control. This could be due to the advanced age of the internode prior to shade treatment, and thus it was less responsive to growth‐promoting factors. There was no consistent response in the growth of leaf blades in response to shading (Figure 3c). The sheaths of the five fully expanded upper leaves in the shade‐treated plants were significantly longer than the sheaths in the control (Figure 3d).

Since the length of the youngest newly‐formed subapical internode in the shade‐treated plants was comparable to the corresponding internode in the control plants (Figure 3b), the identification of differentially expressed genes (DEGs) and associated developmental changes that distinguish these internodes could potentially identify key early regulators promoting internode elongation in response to shade signals. Therefore, the microscopic and RNA‐seq transcriptome studies focused on the youngest newly‐formed subapical internodes of shade‐treated and control plants.

### 2.2 Developmental status of newly‐formed internodes in shade‐treated and control plants

**FIGURE 4:**
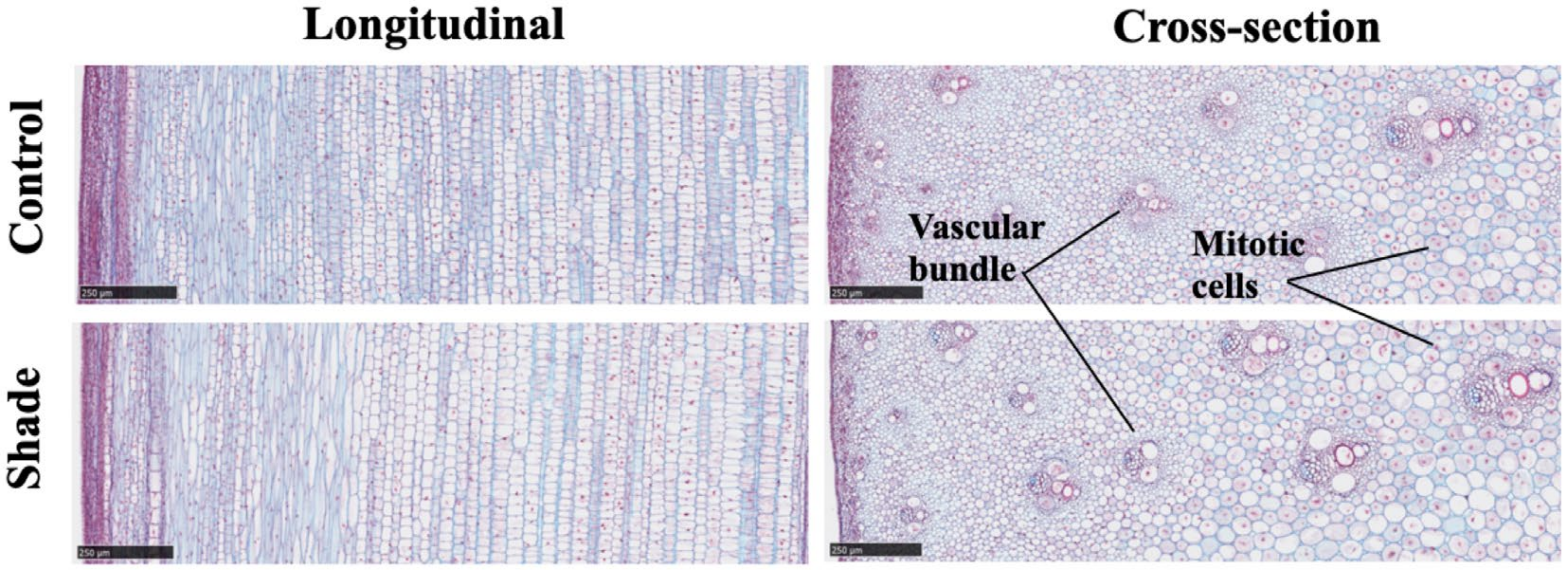
Longitudinal and cross‐section microscopic images of the youngest newly‐formed sub‐apical internodes of bioenergy sorghum genotype R.07020 grown at low plant density (control) or high plant density (shade). The internode samples were stained with Safranin and alcian blue

Longitudinal and cross‐sectional microscopic studies indicate that the youngest subapical internodes in shade‐treated and control plants were at the same developmental stage (Figure 4). The size of the different types of internode cells in shade‐treated and control plants was comparable; the vascular tissues in both were not lignified as shown by the absence of safranin staining of cell walls. Mitotic cells were present in both internode tissues. Therefore, although the internode in the shade‐treated plant at maturity will be at least three times longer than the internode in the control, shade signal had little effect on the developmental status of the internode tissues at their early stages of development.

### 2.3 Transcriptome changes in newly‐formed sorghum internodes in response to shade

Internodes in sorghum and most other grass species develop while enclosed by several layers of leaves and sheath, and as a consequence, internodes are not directly illuminated. Therefore, it is possible that internodes in these species respond to shade signals perceived by leaves that are exposed to direct light that is modified when plants are shaded. The transcriptome of elongated internodes in the shade‐treated plants might be reprogrammed at an earlier stage of development to initiate the shade avoidance response mode of growth. Therefore, we compared the transcriptome of the youngest newly‐formed subapical internodes of shade‐treated and control plants, sampled at 70 DAP (10 days after the onset of the shade treatment), using RNA‐seq. The average length of the youngest newly‐formed supapical internode of shade‐treated and control plants was 1.5 and 1.1 cm, respectively. However, the internode in the shade‐treated plants will eventually become at least three times longer than the internode in the control plants. The RNA‐seq libraries were prepared and sequenced from three shade‐treated plants (independent biological replicates) and three control plants. A total of 33.1 and 35.4 million RNA‐seq reads were generated from the newly‐formed subapical internodes of control and shade‐treated plants, respectively (Table S1). About 90.1% and 91.3% of the reads from the shade‐treated and control plants, respectively, were aligned to the sorghum V3 genome (DOE‐JGI, http://phytozome.jgi.doe.gov/).

Differentially expressed genes in the newly‐formed subapical internode of shade‐treated plants were identified using the following criteria: at least two‐fold higher or lower than the expression level in the corresponding control internode, false discovery rate (FDR) <0.01, and average RPKM ≥2 either in the control or the shade‐treated or both internodes. Prior to analyzing the DEGs between shade‐treated and control internodes, to validate our RNA‐seq data, we compared the transcriptome of the newly‐formed subapical internode of control plants sampled at 70 DAP in the current study with the published transcriptome of the newly‐formed subapical internode at similar developmental stage sampled at 60 DAP (Kebrom et al., 2017). The plants were grown in the same growth chamber under similar conditions at different times. Of 47,205 transcripts, only 23 transcripts were differentially expressed between the internode sampled at 70 DAP and sampled at 60 DAP (Table S2). The fold change of 20 of the 23 DEG is less than threefold. Also, principal component analysis showed that internodes from the control plants sampled at 60 DAP and 70 DAP were more similar to each other and are different from internodes from the shade‐treated plants sampled at 70 DAP (Figure S1). These comparisons confirm the reliability and high quality of our RNA‐seq data.

About 353 transcripts were differentially expressed in internodes of shade‐treated sorghum plants compared to control. The DEGs were annotated using a MapMan mapping file generated using MERCATOR as described in the methods section. About 329 differentially expressed sorghum transcripts were annotated by MapMan (Table S3). A total of 129 genes were upregulated and 200 genes were downregulated in response to shade (Table S4). A higher number of these DEGs function in cell wall metabolism, stress response, regulation of transcription, protein synthesis, plant development, and transport (Table S4). First, we evaluated the expression of sorghum homologs of the Arabidopsis *PHYTOCHROME RAPIDLY REGULATED* (*PAR*) genes such as *ATHB2* and *PIL1* that are rapidly induced by shade (Martinez‐Garcia et al., 2010; Roig‐Villanova et al., 2006). None of the sorghum genes similar to the Arabidopsis *PAR* genes were differentially regulated in the subapical internodes in response to shade.

Shade signals modify the level or activities of plant hormones, such as auxin, BRs, GA, and ethylene, to promote shoot elongation (Kozuka et al., 2010; Stamm & Kumar, 2010). Seven plant hormone‐related genes (MapMan Bin 17) were differentially expressed in response to shade (Tables S3 and S4). Hormone‐related genes downregulated by shade include sorghum gene, *Sobic.010G178500.1*, homologous to the Arabidopsis *Multiprotein Bridging Factor 1* (*AtMBF1c,* at3g24500) gene that functions as a transcriptional coactivator. The expression of *AtMBF1* genes is upregulated by abscisic acid (Tsuda, Tsuji, Hirose, & Yamazaki, 2004), and promote the expansion of cells in leaves (Tojo et al., 2009). The expression of the *MBF1‐like* gene was 29‐fold lower in internodes of shade‐treated sorghum plants (Table S3). A sorghum gene *Sobic.007G156700.1*, homologous to the Arabidopsis *Ethylene Response Factor* (*ERF109*), was upregulated in shade‐treated plants. Arabidopsis ERF109 promotes vascular cell division (Etchells, Provost, & Turner, 2012). Interestingly, sorghum gene *Sobic.002G190700.2*, homologous to Arabidopsis *CycD1* (at1g70210), was expressed fivefold higher in the sorghum internode in response to shade (Table S3). The D‐type cyclins (CycD) promote the transition from the G1 to S phase of the mitotic cell cycle (Dewitte & Murray, 2003).

Shoot elongation in response to shade is also associated with an increase in the expression of cell wall loosening genes which promote cell elongation (Sasidharan, Keuskamp, Kooke, Voesenek, & Pierik, 2014). Of the 15 differentially expressed cell wall–related transcripts, 14 were downregulated in the first subapical internodes of shade‐treated plants (Tables S3 and S4). Most of the downregulated genes encode pectin lyase‐like superfamily proteins and expansins. These genes function in cell wall loosening (Cosgrove, 2016). In Arabidopsis, genes encoding cell wall loosening enzymes such as pectinesterases and pectin‐lyases were upregulated at later stages in response to shade (Devlin, Yanovsky, & Kay, 2003).

To further identify molecular pathways associated with internode elongation in response to shade, we performed gene ontology (GO) enrichment analysis of the differentially expressed sorghum transcripts using the corresponding Arabidopsis gene IDs (Table S3). As shown in Table 1, GO terms for circadian rhythm (GO:0007623), regulation of circadian rhythm (GO:0042752), and GO terms for various stress responses such as response to hydrogen peroxide (GO:0042542), response to salt stress (GO:0009651), protein folding (GO:0006457), and response to water deprivation (GO:0009414) were overrepresented. It is possible that the growth response of the sorghum internodes in response to shade is linked to differential expression of the clock genes. Therefore, we looked at the patterns of expression and function of the differentially expressed clock‐related genes in more detail.

**TABLE 1:** Gene ontology (GO) enrichment analysis of genes differentially expressed in response to shade in the youngest newly‐formed sub‐apical internode of bioenergy sorghum R.07020. Go terms with FDR values < 0.01 were selected for further analysis

GO Accession	GO biological process	*p*‐value	FDR
GO:0007623	Circadian rhythm	1.86E‐06	5.84E‐04
GO:0042752	Regulation of circadian rhythm	1.66E‐07	8.22E‐05
GO:0042542	Response to hydrogen peroxide	4.60E‐07	1.96E‐04
GO:0010555	Response to mannitol	4.76E‐05	5.91E‐03
GO:0006457	Protein folding	1.48E‐05	2.21E‐03
GO:0009414	Response to water deprivation	2.04E‐06	6.07E‐04
GO:0009651	Response to salt stress	6.08E‐08	3.62E‐05
GO:0006355	Regulation of transcription, DNA templated	1.06E‐05	1.75E‐03

Abbreviation: FDR, false discovery rate.

### 2.4 Differential expression of circadian clock genes in sorghum internodes in response to shade

**FIGURE 5:**
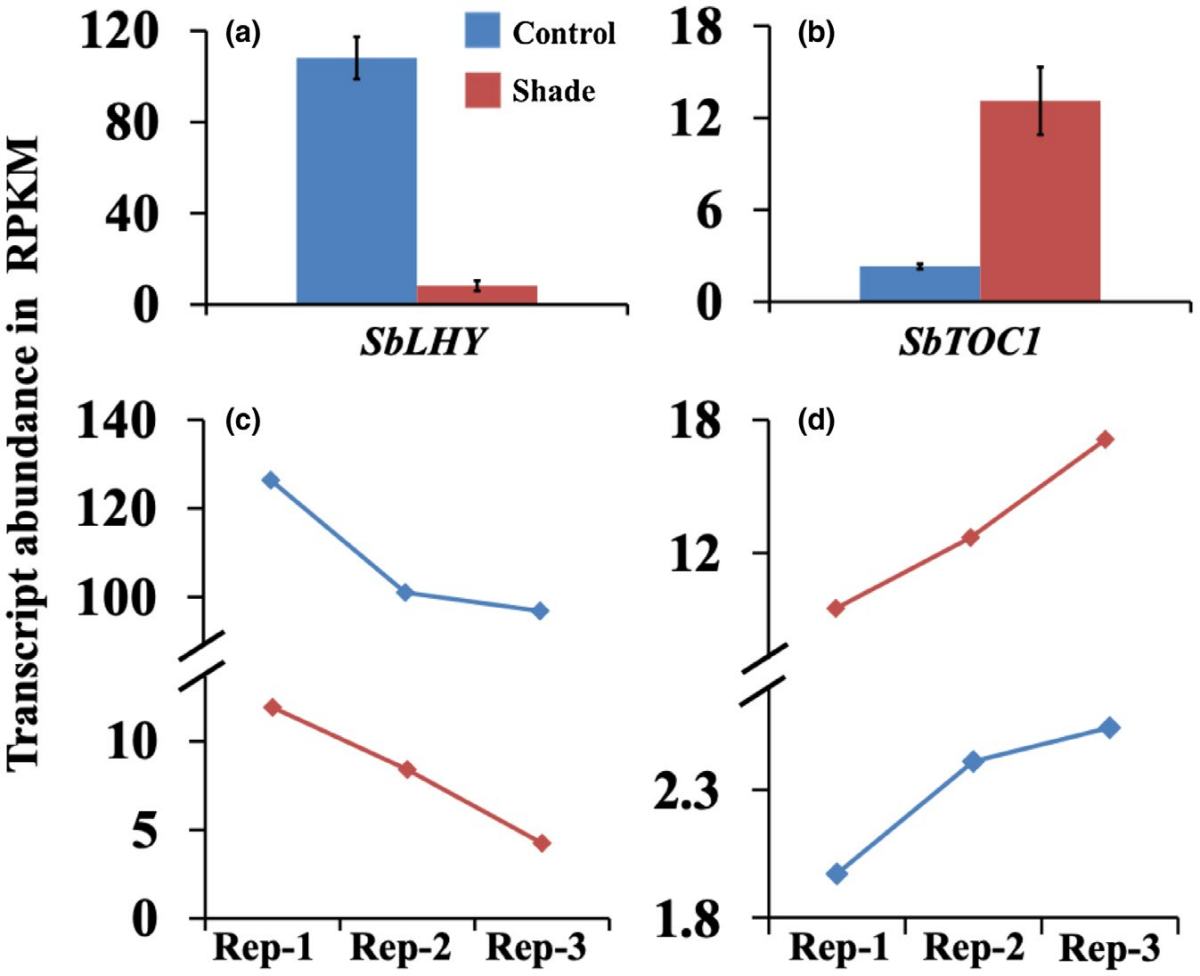
Transcript abundance of core clock morning‐expressed gene *SbLHY* (a) and evening‐expressed gene *SbTOC1* (b) in the youngest newly‐formed sub‐apical internodes of control and shade treated bioenergy sorghum inbred R.07020 plants. The stem internodes were sampled between 4 and 6 hr after the start of the light period (14 hr light/10 hr dark) (Data are mean ± SE; *N* = 3). The first biological replicates (Rep‐1) from control and shade treated plants were harvested at 4 hr after the start of the light period. The second (Rep‐2) and the third (Rep‐3) biological replicates were harvested at 5 and 6 hr after the start of the light period, respectively. (c) Trends in the expression of *SbLHY* in the control and shade treated plants; and (d) trends in the expression of *SbTOC1* in the control and shade treated plants

**FIGURE 6:**
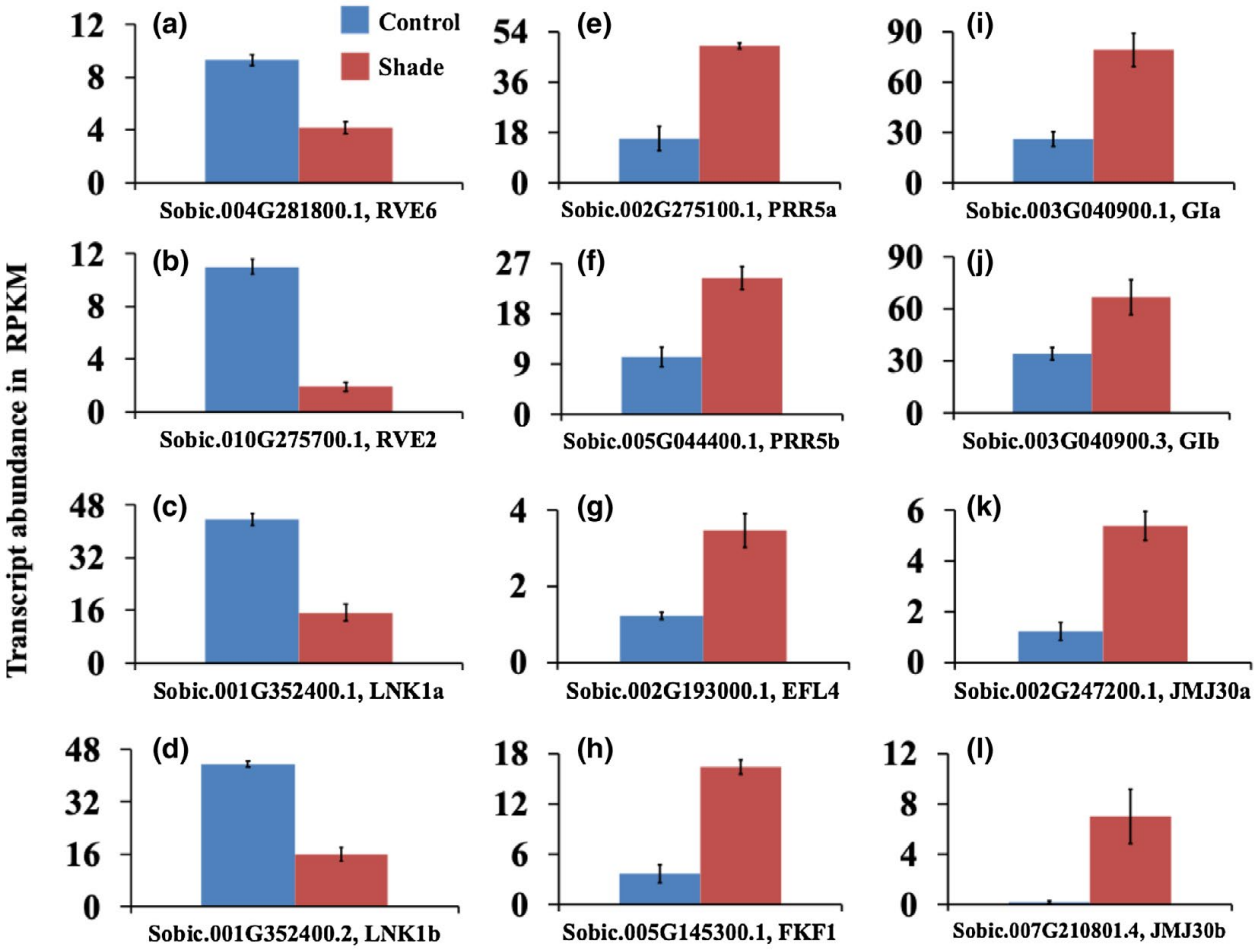
Transcript abundance of clock associated genes in the youngest newly‐formed sub‐apical internodes of control and shade treated bioenergy sorghum R.07020 plants. The internodes were sampled between 4 and 6 hr after the start of the light period (14 hr light/10 hr dark). (a–d) Genes with peak expression in the morning; and (e–l) genes with peak expression in the late afternoon or in the evening. Data are mean ± SE; *N* = 3

**FIGURE 7:**
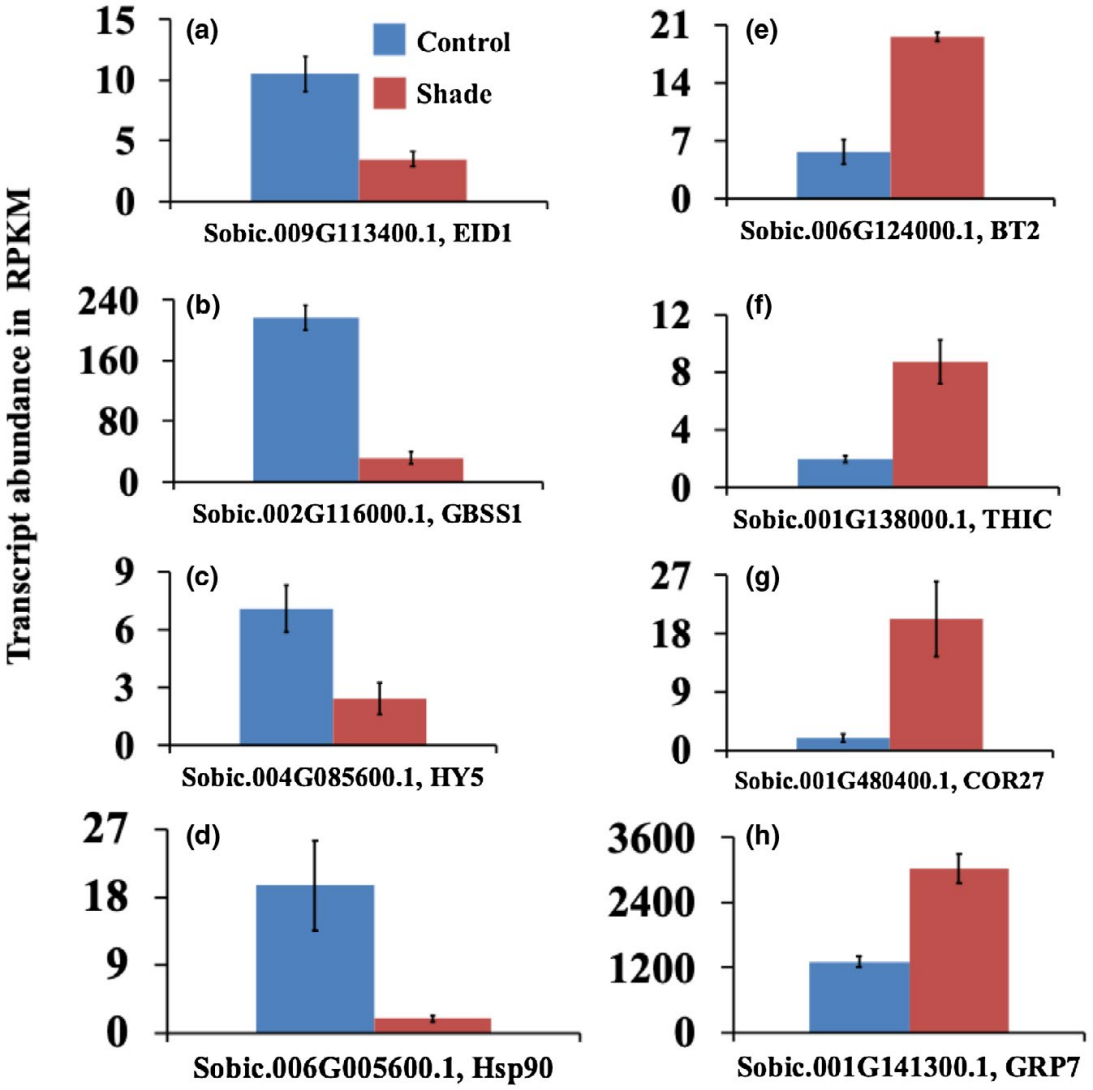
Transcript abundance of genes that function in the input‐pathway or output‐pathway of the clock or clockregulated genes in the youngest newly‐formed sub‐apical internodes of control and shade treated bioenergy sorghum R.07020 plants. The internodes were sampled between 4 and 6 hr after the start of the light period (14 hr light/10 hr dark). (a–d) Genes with peak expression in the morning; and (e–h) genes with peak expression in the late afternoon or in the evening. Data are mean ± SE; *N* = 3

Of the 329 differentially expressed transcripts in the newly‐formed subapical internode of bioenergy sorghum in response to shade annotated by MapMan, at least 24 are core circadian clock, clock‐associated, or clock‐regulated transcripts (Figures 5, 6, 7). The expression of the plant circadian clock genes has been investigated in detail in the model plant Arabidopsis (McClung, 2019). The core oscillator of the Arabidopsis clock is formed by a negative feedback loop of two morning expressed Myb‐like transcription factors, *CIRCADIAN CLOCK ASSOCIATED 1* (*CCA1*) and *LATE ELONGATED HYPOCOTYL* (*LHY*), and evening expressed *PSEUDO‐RESPONSE REGULATOR1* (*PRR1*), which is also known as *TIMING OF CAB EXPRESSION* (*TOC1*). The expression of the sorghum homolog of Arabidopsis *LHY* (*SbLHY*, *Sobic.007G047400.1*) was 12.7‐fold lower and the expression of the sorghum ortholog of *TOC1* (*SbTOC1*, *Sobic.004G216700.1*) was 5.9‐fold higher in the first subapical internodes of shade‐treated plants relative to the corresponding internodes in the control plants (Figure 5a,b). In Arabidopsis, the peak phase of expression of *LHY* is at dawn and the mRNA abundance is progressively reduced during the day; *TOC1* expression starts during the late afternoon and its peak phase is at dusk (De Caluwe et al., 2016). In the current study diurnal expression of *SbLHY* and *SbTOC1* was not investigated. However, the three internodes (biological replicates) from each of the shade‐treated and control sorghum plants were sampled between 4 and 6 hr after the start of the light period (14 hr light and 10 hr dark); one plant from each of control and shade‐treated plants was sampled at every hour. As shown in Figure 5, the expression trend of *SbLHY* was decreasing (Figure 5c) and that of *SbTOC1* was increasing (Figure 5d) from the first replicate sampled at 4 hr after the start of the light period to the third replicate sampled at 6 hr after the start of the light period. The expression trend of the *SbLHY* and *SbTOC1* is consistent with the expression pattern of *LHY* and *TOC1* in Arabidopsis and other plant species. Differential expression of *SbLHY* and *SbTOC1* early during the light period suggests that these genes play a role in regulating internode elongation in response to shade in the bioenergy sorghum plants.

Besides *LHY* and *TOC1*, several other morning and evening expressed Arabidopsis clock genes that form interconnected loops regulate the core clock loop (Hsu & Harmer, 2014). Several sorghum homologs of these clock‐associated genes were differentially expressed in the newly‐formed subapical internode of bioenergy sorghum in response to shade (Figure 6, Table S3). These include downregulation of two morning expressed genes *Sobic.004G281800.1* (*SbRVE6*) and *Sobic.010G275700.1* (*SbRVE2*) homologous to the Arabidopsis *REVEILLE* genes *RVE6* and *RVE2*, respectively (Figure 6a,b). The *REVEILLE* genes belong to a family of Myb‐like transcription factors which include *CCA1* and *LHY* (Rawat et al., 2009; Sanchez & Kay, 2016). *RVE6* is homologous and functionally similar to *RVE4* and *RVE8*, and *RVE2* is similar to *RVE1* and *RVE7* (Rawat et al., 2011). The peak expression of *RVE8* in Arabidopsis precedes dawn and the *rve8* mutant develops a longer hypocotyl when grown in low or medium fluence rate (Rawat et al., 2011). The shoots of triple *rve4 6 8* Arabidopsis seedlings and adult plants are larger than the wild‐type indicating the *RVE8*‐like genes suppress growth (Gray, Shalit‐Kaneh, Chu, Hsu, & Harmer, 2017). Therefore, the downregulation of the *SbRVE6* gene in the sorghum internodes in response to shade might contribute to the enhanced elongation growth of the sorghum stem internodes. The peak circadian expression of *RVE2*, and its homologs *RVE1* and *RVE7*, is before dawn (Rawat et al., 2009). *RVE2* expression is induced by light and repressed by overexpression of *CCA1*, a homolog of *LHY* and *RVE8* (Zhang et al., 2007). *RVE2* mutants flower earlier while their hypocotyl growth is not different from wild‐type (Zhang et al., 2007). However, overexpression of *RVE2* promotes hypocotyl elongation (Rawat et al., 2009). Interestingly, RVE1 and RVE2 promote seed dormancy, and PhyB inhibits their expression and promotes seed germination (Jiang, Xu, Jing, Tang, & Lin, 2016). The downregulation *RVE2* in sorghum internodes in response to shade is distinct from PhyB suppression of *RVE2* during seed germination.

In addition to the *REVEILLE* clock genes, four splice variants of a sorghum *SbLNK1*‐like gene, *Sobic.001G352400.1*, *Sobic.001G352400.2*, *Sobic.001G352400.3*, and *Sobic.001G352400.4* (Table S3), homologous to the Arabidopsis *NIGHT LIGHT‐INDUCIBLE AND CLOCK‐REGULATED 1* (*LNK1*) morning gene (Rugnone et al., 2013), were downregulated in the sorghum internode in response to shade (the expression of two splice variants is shown in Figure 6c,d). The LNK in Arabidopsis interacts with RVE8 to control the circadian expression of core clock genes such as *TOC1* (*PRR1*) and *PPR5* (Ma, Gil, Grasser, & Mas, 2018). The expression of *LNK1* is repressed by TOC1 and PRR5 (Rugnone et al., 2013). The expression of *LNK1* is induced by light pulse in the middle of the night in the wild‐type and *cry1;cry2* double‐mutant Arabidopsis plants, but not in *phyA;phyB* double mutant indicating phytochromes are required for the expression of *LNK1* (Rugnone et al., 2013).

In contrast to the downregulation of morning genes, several circadian clock and clock‐associated evening‐phased genes, in addition to *SbTOC1*, were upregulated in the sorghum internode in response to shade (Figure 6, Table S3). These include upregulation of two sorghum genes, *Sobic.002g275100.1* (*SbPRR5a*) and *Sobic.005G044400.1* (*SbPRR5b*), homologous to the Arabidopsis *PRR5* gene (Figure 6e,f). PRR5 in Arabidopsis represses the expression of morning genes including *CCA1* and *LHY* (Nakamichi et al., 2010). However, the hypocotyl of *prr5* mutant is longer than the wild‐type (Ito et al., 2008). The sorghum gene, *Sobic.002g193000.1*, homologous to the Arabidopsis *ELF4‐LIKE4* (*EFL4*), was upregulated in the shade‐treated internode (Figure 6g). The function of EFL4 in Arabidopsis is not yet known. However, its homologous gene ELF4 is a component of the circadian clock with peak expression in the evening and functions in the regulation of hypocotyl elongation and flowering time (Doyle et al., 2002; McWatters et al., 2007). A sorghum gene (*SbFKF1, Sobic.005G145300.1*) homologous to the Arabidopsis *FLAVIN‐BINDING, KELCH REPEAT,* and *F BOX 1* (*FKF1*) gene was upregulated in the shad‐treated internode (Figure 6h). The expression of *FKF1* in Arabidopsis is controlled by the circadian clock, with peak expression in the late afternoon (Nelson, Lasswell, Rogg, Cohen, & Bartel, 2000). Two splice variants of a sorghum gene (*SbGI*, *Sobic.003G040900.1,* and *Sobic.003G040900.3*) homologous to the Arabidopsis *GIGANTEA* (*GI*) gene were upregulated in the shade‐treated internode (Figure 6i,j). The Arabidopsis *GI* gene is expressed in the evening and synchronizes the pace of the clock with the prevailing environmental conditions, and mediates the response to light signaling and photoperiodic flowering time (Fowler et al., 1999; Huq, Tepperman, & Quail, 2000; Martin‐Tryon, Kreps, & Harmer, 2007; Mizoguchi et al., 2005). Two sorghum genes (*Sobic.002G247200.1* and *Sobic.007G210801.4*) homologous to the Arabidopsis *JUMONJI C DOMAIN‐CONTAINING PROTEIN* (*JMJ30, JMJ5*) were upregulated in the shad‐treated internode (Figure 6k,l). The *JMJ30* gene in Arabidopsis is expressed in the evening and controls the period length (Lu et al., 2011). The morning genes CCA1 and LHY repress the expression of *JMJ30* (Lu et al., 2011).

### 2.5 Differential expression of circadian clock‐associated or clock‐controlled genes in sorghum internodes in response to shade

Several clock‐associated or clock‐controlled genes were differentially expressed in sorghum internodes in response to shading (Figure 7, Table S3). The expression of genes associated with morning core clock genes was downregulated and the expression of genes associated with evening core clock genes was upregulated. A sorghum gene, *Sobic.009G113400.1*, homologous to the *EID1* gene of Arabidopsis was downregulated by shade (Figure 7a). The *EID1* gene functions in phytochrome signaling and light input pathway to the core clock (Dieterle, Zhou, Schafer, Funk, & Kretsch, 2001; Muller, Zhang, Koornneef, & Jimenez‐Gomez, 2018). A mutant *EID1* allele in cultivated tomato selected during domestication from wild ancestors reduced the speed of the clock and enabled the cultivation of tomato in higher latitudes (Muller et al., 2016). A sorghum gene, *Sobic.002G116000.1*, homologous to the Arabidopsis *Granule Bound Starch Synthase1* (*GBSS1*) gene that functions in starch biosynthesis was 6.6‐fold lower in the shade‐treated internode (Figure 7b). The expression of *GBSS1* is regulated by CCA1 and LHY, with peak expression in the morning (Moraes et al., 2019; Ortiz‐Marchena et al., 2014; Tenorio, Orea, Romero, & Merida, 2003). A sorghum gene (*SbHY5*, *Sobic.004G085600.1*) homologous to the Arabidopsis *ELONGATED HYPOCOTYL* 5 (*HY5*) was downregulated in the sorghum internode in response to shade (Figure 7c). The *HY5* gene in Arabidopsis encodes a basic leucine zipper (bZIP) transcription factor that regulates the expression of many genes that function in diverse physiological and developmental processes, including photomorphogenesis, circadian clock, light and hormone signaling, and cell elongation (Gangappa & Botto, 2016). The expression of *HY5* is regulated by light perceived by the plant photoreceptors. HY5 functions as a signaling molecule that transduces the R:FR light status to roots (Chen et al., 2016; van Gelderen et al., 2018). In addition, HY5 transduces red and blue light signals to the core clock (Hajdu et al., 2018).

About 30 differentially expressed transcripts were assigned to the stress bin (Bin 30) of the MapMan (Table S4). Six of these genes were upregulated and 24 downregulated in the first subapical stem internode of bioenergy sorghum in response to shade (Table S4). About 14 of those downregulated sorghum transcripts are homologs to the Arabidopsis genes encoding heat‐shock proteins (Hsp) (Table S3). Some of the Arabidopsis Hsp genes function in the circadian clock (McClung, 2019). The Arabidopsis heat‐shock proteins Hsp40, Hsp70, and Hsp90 are involved in the maturity and stability of the F‐box protein ZEITLUPE (ZTL). ZTL ubiquitylates the evening genes TOC1 and PRR5 at night and targets them to degradation by the proteasome, which shortens the period. In addition, it appears that cytosolic Hsp90 proteins contribute to the entrainment of the Arabidopsis circadian clock through their action on the morning genes *CCA1*/*LHY* (Davis et al., 2018). Interestingly, the expression of the sorghum gene, *Sobic.006G005600.1*, homologous to the Arabidopsis Hsp90, was 9.6‐fold lower in stem internodes of R.07020 sorghum plants in response to shade (Figure 7d). In addition, the expression of three sorghum transcripts, *Sobic.006G055600.1*, *Sobic.003G350700.1*, and *Sobic.009G163900.1*, similar to the Arabidopsis Hsp70 were downregulated in the subapical stem internodes in response to shade (Table S3).

A sorghum gene (*SbBT2*, *Sobic.006G124000.1*) homologous to the Arabidopsis *BTB AND TAZ DOMAIN PROTEIN 2* (*BT2*) was upregulated in the shade‐treated internode (Figure 7e). The *BT2* gene is expressed in the evening and its expression is controlled by the circadian clock (Mandadi, Misra, Ren, & McKnight, 2009). The *BT2* gene mediates response to hormones, sugar, and nutrients (Mandadi et al., 2009). Another evening expressed sorghum gene, *Sobic.001G138000.1*, homologous to the Arabidopsis *THIAMIN C SYNTHASE* (*THIC*) gene that encodes a protein involved in thiamin biosynthesis was upregulated in the newly‐formed internode of shade‐treated sorghum plants (Figure 7f). The expression of *THIC* is regulated by the circadian clock and its expression peak is at the end of the light period, similar to the peak expression of evening genes such as *TOC1*, and repressed during the day by the morning genes *CCA1* and *LHY* (Bocobza et al., 2013). Consistent with this, a binding site for the CCA1 and LHY was found in the promotor of *THIC* (Bocobza et al., 2013). A sorghum gene, *Sobic.001G480400.1*, homologous to the Arabidopsis *COLD REGULATED GENE 27* (*COR27*) was upregulated in the newly‐formed sorghum internodes in response to shade signals (Figure 7g). COR27 promotes flowering and reduces freezing tolerance (Li et al., 2016). The expression of COR27 is repressed by the morning gene *CCA1* (Li et al., 2016; Wang et al., 2017). The peak expression of *COR27* is in the evening and represses the expression of *TOC1* and *PRR5* (Li et al., 2016; Wang et al., 2017). A sorghum gene, *Sobic.001G141300.1*, similar to the Arabidopsis *AtGRP7*, was upregulated in internodes of shade‐treated sorghum plants (Figure 7h). The evening expressed gene *AtGRP7* functions as a component of the output pathway of the circadian clock (Heintzen, Nater, Apel, & Staiger, 1997; Schmal, Reimann, & Staiger, 2013).

In summary, RNA‐seq transcriptome analysis revealed differential expression of circadian clock genes in the newly‐formed subapical sorghum internodes in response to shade. Interestingly, shading downregulated the expression of morning genes and upregulated the expression of evening genes around 4 hr after the start of the 14‐hr‐long light period.

## 3 DISCUSSION

Bioenergy sorghum plants produce a large amount of biomass, which is mainly accumulated in the stem internodes (Olson et al., 2012). Understanding the physiological and molecular mechanisms controlling stem growth in bioenergy sorghum will help to modify stem growth and biomass accumulation of crops as desired. Therefore, we investigated the growth of stem internodes in the bioenergy sorghum inbred R.07020. Our study revealed that internode elongation in R.07020 during the vegetative phase is a response to high plant density, a typical growth response of plants anticipating shading by their neighbors known as the shade avoidance response (Casal, 2013; Martinez‐Garcia et al., 2010; Smith & Whitelam, 1997). It appears that this phenomenon remained unnoticed in the bioenergy sorghum R.07020 because like any other agronomic crop it is grown in the field at high plant density. However, by changing planting density in the field and growth chambers, we discovered that internode elongation in R.07020 plants is a response to mutual shading at high planting density, and stem elongation is suppressed when the plants are grown at low planting density in the absence of shade signals from neighbor plants.

Internodes in sorghum are initiated sequentially from the rib zone of the shoot apical meristem and transition through different developmental stages until they become fully elongated and stop growing. As in maize, the first four subapical internodes in sorghum plants are at different developmental stages, from the youngest newly‐formed first subapical internode to the more mature and longer fourth subapical internode (Kebrom et al., 2017; Morrison, Kessler, & Burton, 1994). As shown in Figure 3, the first subapical internodes in shade‐treated and control plants were comparable in length. In addition, microscopic analysis of the stem tissues indicates that the internodes were at similar developmental stages. However, at maturity, the length of the internode in the shade‐treated plants will be at least three times longer than the internode in the control. We hypothesized that key genes that initiate internode elongation in response to shade might be activated in the newly‐formed subapical internode before any visible change in growth. Therefore, to identify the molecular mechanisms that regulate the early events of internode elongation in response to shade, we analyzed the transcriptome of the first newly‐formed subapical internodes in shade‐treated and control sorghum plants.

Molecular mechanisms that enhance shoot elongation in response to shade signals have been investigated in more detail in younger plants of Arabidopsis and other species. Several *PAR* genes that are markers of shade signaling such as *ATHB2* and *PIL1* are rapidly upregulated in response to shade in these species (Ciolfi et al., 2013; Procko et al., 2014). Unlike the detailed study in young plants, the response of adult plants to shade has not been studied in detail (Nozue et al., 2015). Furthermore, in sorghum and other monocots, the molecular mechanisms promoting stem elongation in response to shade could be different from eudicots because the stem internodes in sorghum grow while enclosed by several layers of young leaves and sheaths, reducing exposure to direct light. Consistent with these PAR genes that are markers of perception of shade signals were not among the 353 DEGs in the sorghum internodes in response to shade. The results indicate that shade signals in bioenergy sorghum might be perceived by leaves and indirectly promote internode elongation.

Hypocotyl elongation in response to shade signals in Arabidopsis seedlings involves inactivation of PhyB, which allows PIFs to transcribe auxin biosynthesis genes (Hornitschek et al., 2012; Lorrain et al., 2008; Muller‐Moule et al., 2016). An increase in auxin production promotes cell elongation, and thus hypocotyl elongation, through increasing the expression of cell wall loosening genes such as *xyloglucan endotransglucosylase hydrolase* (*XTH*) (Sasidharan et al., 2010). In the newly‐formed subapical sorghum internodes, there was little change in the expression of hormone biosynthesis or signaling genes in response to shade. In addition, shading downregulated several cell wall loosening genes in sorghum internodes. It is likely that differential expression of hormone biosynthesis and signaling genes occurs once the internode starts to elongate. However, the downregulation of cell wall loosening genes, the upregulation of *ERF109‐like* gene that promotes vascular cell division (Etchells et al., 2012), and the downregulation of *MBF1‐like* gene that promotes cell expansion in Arabidopsis leaves (Tojo et al., 2009) suggest internode elongation in response to shade in the bioenergy sorghum R.07020 is associated with an increase in cell division and/or a delay in cell expansion. Consistent with this, the expression of a sorghum gene homologous to the Arabidopsis *CycD1* gene was upregulated in response to shade. Interestingly, a mutation in the Arabidopsis *CycD1* gene delays the onset of cell division (Masubelele et al., 2005). And, the *CycD1* in *Antirrhinum majus* accelerates entry into the mitotic cell cycle and rate of growth (Koroleva et al., 2004). Organ growth in plants is due to both cell division and cell elongation (Beemster & Baskin, 1998). Therefore, internode elongation in response to shade in bioenergy sorghum could be in part due to an increase in the rate and phase of cell division leading to a higher cell number in the elongated internodes.

Differential expression of circadian clock and clock‐associated genes indicates the early molecular events promoting internode elongation in response to shade in the bioenergy sorghum R.07020 involves alterations in the activities of the circadian clock. Interestingly, the expression of sorghum genes homologous to the Arabidopsis morning genes *LHY*, *RVE6*, *RVE2*, and *LNK* was downregulated, and evening genes *TOC1*, *PRR5*, and *GI* was upregulated in the sorghum internode in response to shade. Also, the expression patterns of genes that function in the input and output pathways of the clock were similar to the expression patterns of the core clock genes; morning expressed genes were downregulated and evening expressed genes were upregulated (Figure 7). The Arabidopsis core clock genes *CCA1*/*LHY* and *TOC1* reciprocally inhibit each other's expression (McClung, 2019). It is possible that, in the bioenergy sorghum internodes, shade signals indirectly inhibited the expression of key morning expressed genes such as *LHY* during the early hours of the morning, and thus the evening genes were released from repression. Also, it is possible that shade signals shifted the phase of the clock in the sorghum internodes. This is consistent with far‐red induced phase shift of the clock in Arabidopsis seedlings (Wenden et al., 2011; Yanovsky, Mazzella, Whitelam, & Casal, 2001).

There are a few studies that indicate a role for the plant circadian clock in gating enhanced shoot growth in response to shade signals. For example, Sellaro, Pacin, and Casal (2012) reported that shade signal applied in the morning did not promote shoot elongation in Arabidopsis; whereas shade signal applied in the afternoon, when the expression of *LHY and CCA1* was low, promoted shoot elongation. In the *lhy‐cca1* mutant, shoot elongation was promoted by FR light treatment applied in the morning as well as in the afternoon. However, unlike in Arabidopsis, the expression of morning genes such as *LHY* in the sorghum internodes was downregulated by shade. Differential expression of circadian clock genes in response to shade in sorghum internodes provide additional evidence for the role of the circadian clock in mediating shoot elongation in response to shade.

Noticeable differential expression of circadian clock genes has not been reported in transcriptome studies of SAS in Arabidopsis seedlings and in the stem of tomato and other species (Cagnola, Ploschuk, Benech‐Arnold, Finlayson, & Casal, 2012; Devlin et al., 2003). However, morning genes were downregulated and evening genes were upregulated in elongating maize seedlings grown with simulated shade (Wang et al., 2016). Unlike in the current study in sorghum internodes, shade signal marker genes such as *ZmHB53*, which is an ortholog of the Arabidopsis *ATHB2* gene, were also differentially expressed in the maize seedlings (Wang et al., 2016). It is likely that plant tissues sampled for the transcriptome analysis of maize seedlings were composed of leaves exposed to light and young tissues enclosed with leaves and sheath. In addition, Salter, Franklin, and Whitelam (2003) showed that the expression of *PIL1*, a shade marker gene, was upregulated in the morning in response to shade, while maximal elongation response was documented in the evening when the evening clock gene *TOC1* was expressed. The authors concluded that both PIL1 and TOC1 are required for elongation in response to shade. Therefore, the regulation of the circadian clock and response to shade in plant tissues that are exposed to light could be different from tissues that are not exposed to light. In tissues that are illuminated with direct light, the simultaneous effect of photoreceptors on shade signaling and clock resetting in the morning might suppress the role of the clock in promoting shoot elongation in response to shade. Consistent with this, it appears that maximal shoot elongation in response to shade in Arabidopsis is delayed until in the afternoon when the level of morning genes is reduced (Sellaro et al., 2012). In sorghum, shade signals promoted internode and sheath elongation but did not increase the growth of leaf blades that are illuminated (Figure 3). Interestingly, the sheath in sorghum also grows partially enclosed by an older sheath. It is possible that the enclosed section of the sheath, and not the illuminated section, elongated in response shade.

A link between enhanced shoot growth and reduced expression of *CCA1‐like* gene, which is a homolog of *LHY*, has been reported in maize (Ko et al., 2016). Interestingly, the *CCA1* gene in maize is homologous to the *LHY* gene of sorghum identified among DEGs in the current study. The ears in maize develop while they are enclosed with leaves and may not perceive light. The morning genes such as *CCA1* and *LHY* were downregulated at all times in maize ears (Hayes et al., 2010). Interestingly, while the expression of the *LHY* gene in maize ears was low over a 24 hr period, the cycling of morning genes with lower amplitude has been documented. In the current study, we did not determine the circadian expression of clock genes in stem internodes. However, there was a decreasing trend in the expression of *LHY* and an increasing trend in the expression of *TOC1* in stem internodes of shade‐treated and control plants sampled between 4 hr and 6 hr after the start of the light period. The results indicate the similarity in the patterns and levels of expression of clock genes between ears of maize and internodes of shade‐treated sorghum plants.

## 4 CONCLUSION

The current study identified that shade signals promote internode elongation in the bioenergy sorghum genotype R.07020. Since internodes in bioenergy sorghum grow while enclosed by leaves, we hypothesize that shade signals indirectly alter the function or patterns of expression of the circadian clock genes to accelerate internode elongation. In tissues that are not illuminated, it is possible that the expression of clock genes is modulated by other factors. For example, sucrose can modulate the function of the circadian clock (Dalchau et al., 2011; Philippou, Ronald, Sanchez‐Villarreal, Davis, & Davis, 2019), and downregulation of *CCA1* in Arabidopsis leaves enhanced chlorophyll and starch content and growth vigor (Ni et al., 2009). Therefore, changes in carbohydrate metabolism and partitioning in response to shade signals may alter the patterns and timing of expression of clock genes in stem internodes of bioenergy sorghum.

## 5 MATERIALS AND METHODS

### 5.1 Plant materials and growing conditions

Seed of bioenergy sorghum genotype R.07020 were planted in 3‐gallon pots filled with the commercial growth mix MVP and field soil (3:1) in two growth chambers, 12 pots in each chamber. The growth chambers were supplied with incandescent and florescent lamps producing about 350 µmol m^−2^ s^−1^ photosynthetically active radiation (PAR). The temperature in the growth chambers was 31°C during the light period (14 hr) and 22°C during the dark period (10 hr). Four seeds were planted in each pot and thinned to one seedling per pot at the three‐leaf stage. Tillers were removed when they emerged in order to reduce shading among plants and only the main shoot was maintained. At 60 days after planting (DAP), shade treatment was started by transferring six potted plants from one chamber to the second chamber, and thus the number of plants in one of the growth chambers was 6 (control, low plant density) and the number of plants in the second growth chamber was 18 (shade, high plant density). In addition, the spacing between plants in the shade treatment was reduced by crowding the potted plants to enhance competition for light. Plant height, stem length and the length of the leaf blade, and sheath of the youngest five fully expanded leaves of plants in the control and shade were measured at 10 days after the start of the shade treatment (70 DAP). The first subapical internodes from three independent plants of shade‐treated and control were harvested for RNA‐seq library preparation. Sampling was done between 4 and 6 hr after the start of the light period; one plant from control and one plant from shade‐treated plants were sampled in every hour. The experiment was repeated several times to harvest internode samples for microscopic study and for photographic documentation of whole plants and stem internodes.

### 5.2 Microscopic study of internode samples

Longitudinal and cross‐sections from the median region of the first subapical internodes of shade‐treated and control plants were prepared for microscopic study of the development of the stem tissues. The internode sections were fixed in FAA overnight and stored in 70% ethanol. Subsequent tissue dehydration, embedding in paraffin, sectioning to 10 µm, and staining with alcian blue and safranin were performed at the histopathology laboratory at Texas A&M University School of Veterinary Medicine. The slides were scanned at 20X using Nanozoomer HT digital slide scanner, and the images were viewed using NDP.view2 software (Hamamatsu Photonics).

### 5.3 RNA‐seq library preparation, sequencing, and analyses

To identify the early molecular events that mediate internode elongation in response to shade signals in the bioenergy sorghum inbred line R.07020, RNA‐seq libraries were prepared from the newly‐formed subapical internodes of shade‐treated and control plants. Since the lengths of the newly‐formed subapical internodes of shade‐treated and control plants were less than 2 cm, RNAs were extracted from the whole internodes. RNA‐seq libraries were prepared from three biological replicates. The methods for RNA extraction, RNA‐seq library preparation, sequencing, and analysis are as described in Kebrom et al. (2017). Briefly, RNA from internode samples was extracted using TRIzol (Invitrogen). RNA‐seq libraries were prepared using TruSeq™ RNA Sample Prep Kit v2 according to the recommended protocol (Illumina Inc.). RNA‐seq library quality was evaluated using a Bioanalyzer (Agilent Technologies Inc.) and single‐end reads were sequenced on an Illumina HiSeq2500 at the Texas A&M University Genomics and Bioinformatics Service Center. Principal component analysis of biological replicates of control and shaded samples was performed in R following the method published by CLC Genomics Workbench User Manual (CLC bio). Initially, transcripts with CPM values <2 for any of the nine biological replicates were removed from the analysis. Next, TMM normalization of the retained transcripts was performed to calculate the effective library sizes required for further normalization steps and CPM was again calculated using the TMM effective library sizes. These CPM values were then log‐transformed to obtain normality of the expression distribution. Next, working with logCPM‐normalized data, z‐score normalization was performed across all transcripts for each biological replicate. These z‐scores were used as input into PCA.

To identify DEGs, sequence reads were aligned to the sorghum V3 genome (DOE‐JGI, http://phytozome.jgi.doe.gov/) and statistically analyzed using EdgeR in the CLC bio workbench (CLC bio). A MapMan mapping file was created using Mercator by Blast search of the *sorghum bicolor* V3.1 transcripts against the Arabidopsis proteome, and DEGs were annotated using the MapMan software (Lohse et al., 2014; Thimm et al., 2004). GO overrepresentation test was conducted using the corresponding Arabidopsis IDs of the sorghum DEGs for GO biological process in GO ontology database.

## CONFLICT OF INTEREST

The authors declare that they have no conflict of interest.

## AUTHOR CONTRIBUTION

THK and JEM conceived the research project; THK performed the research; and THK, BAM, and JEM analyzed the data. THK, BAM, and JEM wrote the manuscript. All authors read and approved the final manuscript.

## Supporting information

Table S1‐S4‐FigS1

Table S2

Table S3
