# Supplementary material for: Shade signals alter the expression of circadian clock genes in newly‐formed bioenergy sorghum internodes
Source: Plant Direct. 2020 Jun 25;4(6):e00235. doi: 10.1002/pld3.235 (PMC7315773; doi:10.1002/pld3.235)
Supplement: Supplementary file 1 — Table S1‐S4‐FigS1 [file PLD3-4-e00235-s001.pptx]

## Slide 1
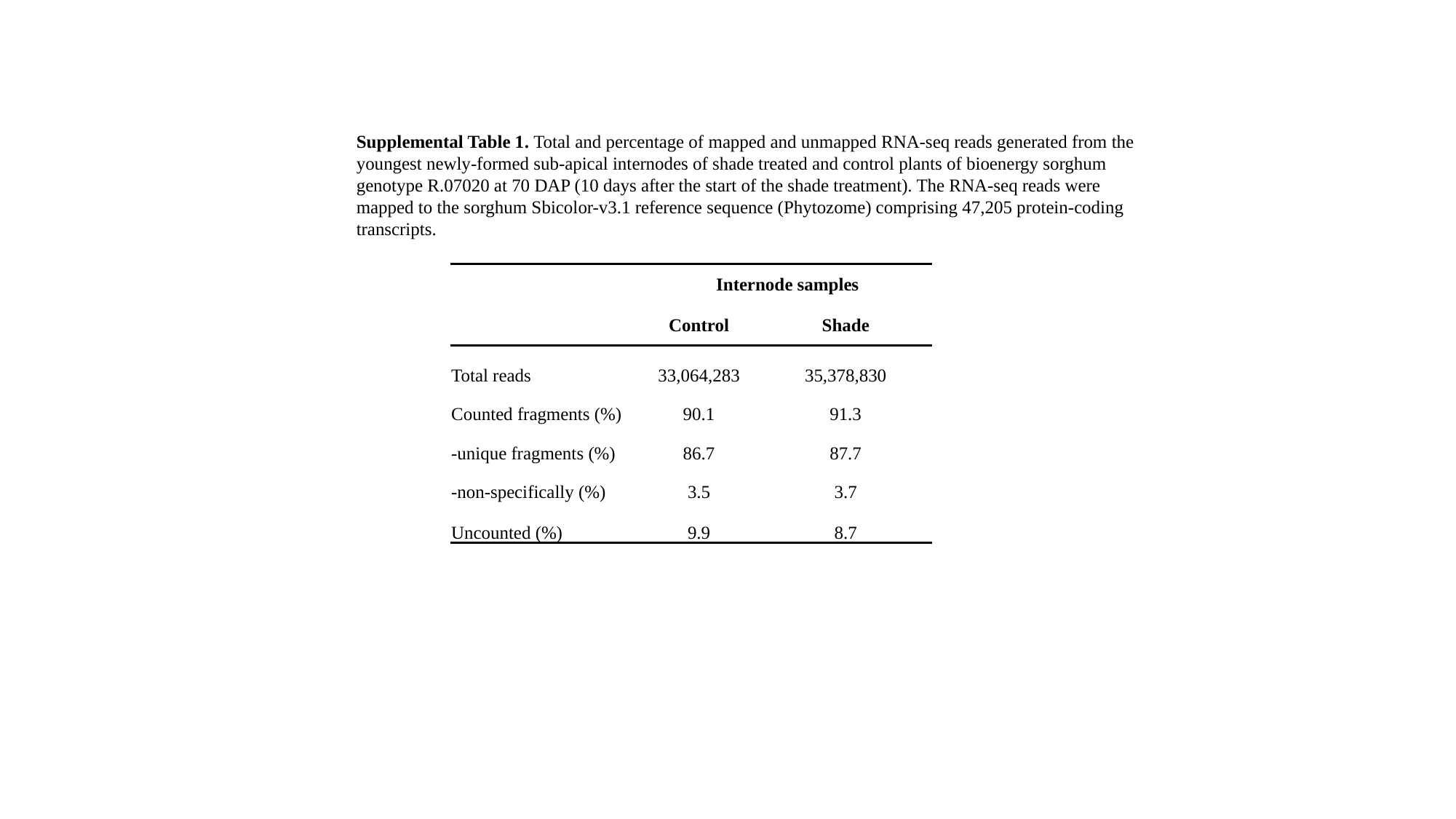

Supplemental Table 1. Total and percentage of mapped and unmapped RNA-seq reads generated from the youngest newly-formed sub-apical internodes of shade treated and control plants of bioenergy sorghum genotype R.07020 at 70 DAP (10 days after the start of the shade treatment). The RNA-seq reads were mapped to the sorghum Sbicolor-v3.1 reference sequence (Phytozome) comprising 47,205 protein-coding transcripts.
| | Internode samples | |
| --- | --- | --- |
| | Control | Shade |
| Total reads | 33,064,283 | 35,378,830 |
| Counted fragments (%) | 90.1 | 91.3 |
| -unique fragments (%) | 86.7 | 87.7 |
| -non-specifically (%) | 3.5 | 3.7 |
| Uncounted (%) | 9.9 | 8.7 |

## Slide 2
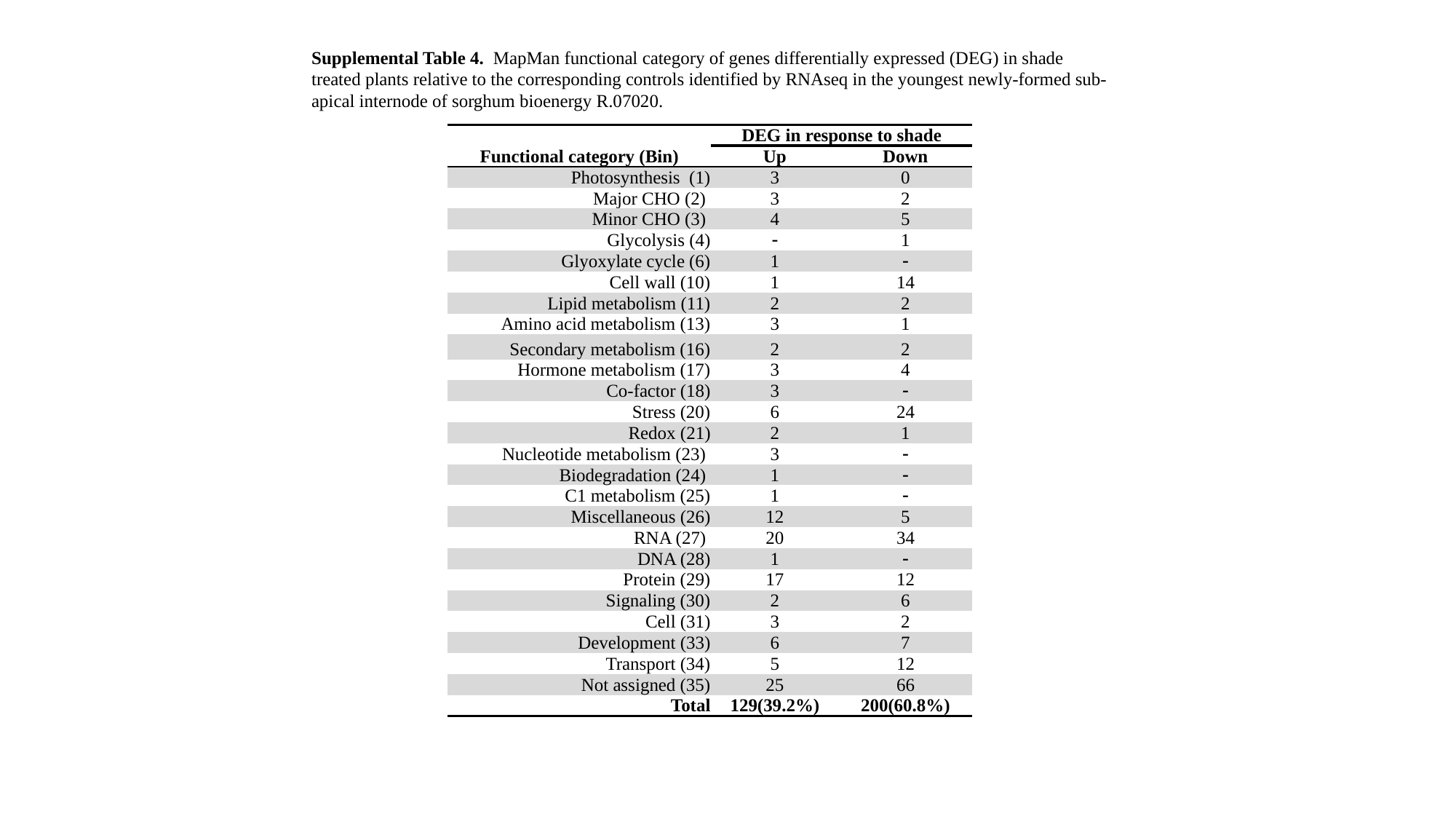

Supplemental Table 4. MapMan functional category of genes differentially expressed (DEG) in shade treated plants relative to the corresponding controls identified by RNAseq in the youngest newly-formed sub-apical internode of sorghum bioenergy R.07020.
| | DEG in response to shade | |
| --- | --- | --- |
| Functional category (Bin) | Up | Down |
| Photosynthesis (1) | 3 | 0 |
| Major CHO (2) | 3 | 2 |
| Minor CHO (3) | 4 | 5 |
| Glycolysis (4) | − | 1 |
| Glyoxylate cycle (6) | 1 | − |
| Cell wall (10) | 1 | 14 |
| Lipid metabolism (11) | 2 | 2 |
| Amino acid metabolism (13) | 3 | 1 |
| Secondary metabolism (16) | 2 | 2 |
| Hormone metabolism (17) | 3 | 4 |
| Co-factor (18) | 3 | − |
| Stress (20) | 6 | 24 |
| Redox (21) | 2 | 1 |
| Nucleotide metabolism (23) | 3 | − |
| Biodegradation (24) | 1 | − |
| C1 metabolism (25) | 1 | − |
| Miscellaneous (26) | 12 | 5 |
| RNA (27) | 20 | 34 |
| DNA (28) | 1 | − |
| Protein (29) | 17 | 12 |
| Signaling (30) | 2 | 6 |
| Cell (31) | 3 | 2 |
| Development (33) | 6 | 7 |
| Transport (34) | 5 | 12 |
| Not assigned (35) | 25 | 66 |
| Total | 129(39.2%) | 200(60.8%) |

## Slide 3
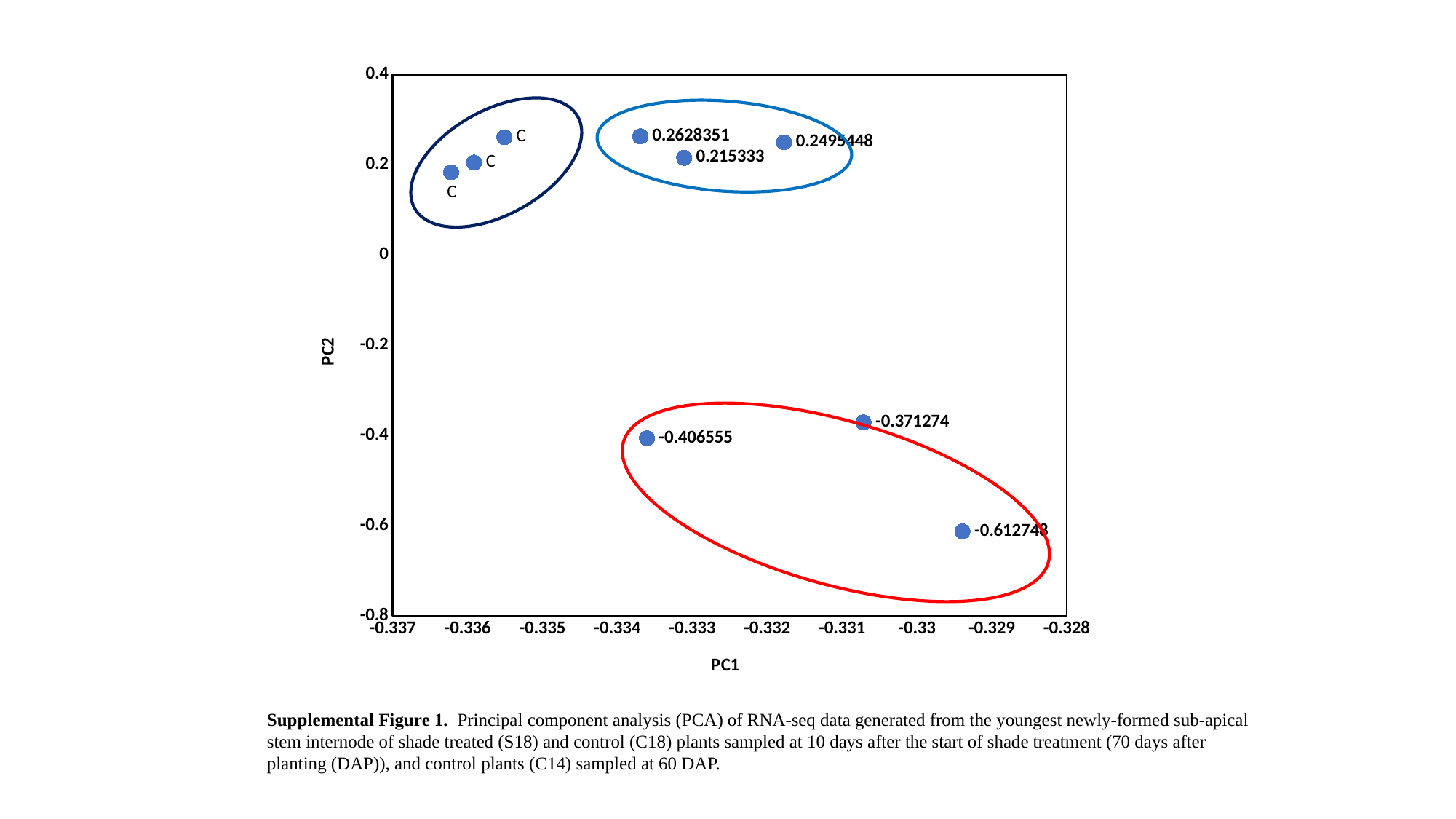

### Chart
| Category | PC2 |
|---|---|
Supplemental Figure 1. Principal component analysis (PCA) of RNA-seq data generated from the youngest newly-formed sub-apical stem internode of shade treated (S18) and control (C18) plants sampled at 10 days after the start of shade treatment (70 days after planting (DAP)), and control plants (C14) sampled at 60 DAP.
